# Frequency and Severity of Adverse Drug Reactions to Medications Prescribed for Alzheimer’s Disease in a Brazilian City: Cross-Sectional Study

**DOI:** 10.3389/fphar.2020.538095

**Published:** 2020-12-15

**Authors:** Tânia Regina Ferreira, Luciane Cruz Lopes, Cristiane de Càssia Bergamaschi

**Affiliations:** Graduate Program of Pharmaceutical Sciences, University of Sorocaba, Sorocaba, Brazil

**Keywords:** Alzheime’s disease, safety, adverse reaction, pharmacoepidemiology, older adults

## Abstract

**Background:** There is lack of national studies that assess the risks associated with the drugs provided under the Brazilian public health system for treating Alzheimer’s disease. Then, this study determined the prevalence and severity of self-reported adverse drug reactions (ADRs) prescribed to patients with Alzheimer’s disease in the Brazilian public health system.

**Methods:** A cross-sectional study was carried out based on public data from the MEDEX system (information on dispensing data, known as exceptional dispensing medications) and interviews with patients and/or caregivers who get access to Alzheimer’s drugs at a public pharmacy in a large Brazilian city, between July and September 2017, inquiring about ADRs and serious adverse events (SAEs).

**Results:** The subjects were asked about ADRs and SAEs related to the use of donepezil, galantamine, rivastigmine and memantine. Out of 285 patients enrolled on the database, 250 participated in the study (87.7%). Among the participants, approximately 63.0% were female, 70.3% aged ≥75 years and 70.3% had comorbidities. Overall, 209 patients (83.6%) reported at least one ADR (total 1,149 ADRs) and rivastigmine was associated with the largest number of ADRs per patient (7.9 ADRs/patient). The predominant adverse effects were psychiatric disorders with common frequency (57.1%) and mild severity (89.0%). Six patients (2.4%) had SAEs that required hospitalization. The use of antipsychotics was the variable associated with ADR (OR = 4.95; 95% CI: 1.45–16.93; *p* = 0.011).

**Conclusion:** There was a large number of reported ADRs and most of them were of common frequency and mild severity, being mainly related to psychiatric disorders. Considering the fragility of these patients, it is important to improve safety-related care in the use of drugs for treating this disease.

## Introduction

Dementia is characterized by a cognitive decline that significantly impacts a person’s ability to perform activities of daily living. Alzheimer’s disease, the most common form of dementia, has become a major health problem worldwide, as the number of older adults continues to rise (Winblad et al., 2016). The number of older adults suffering from Alzheimer’s disease totals 35.6 million globally and, by 2,050, this figure is set to increase to 115.4 million (Prince et al., 2013).

Brazil´s population is around 210,158,000, consisting of approximately 20 million (9.5%) older adults aged 65 and above. Over the next few decades, the proportion of older adults will double and account for 21.9% of the total population (Instituto Brasileiro de Geografia e Estatística IBGE, 2019). However, determining dementia prevalence in the Brazilian population is difficult due to the regional nature of the studies available, which may not be representative of the country as a whole (Fagundes et al., 2011).

A systematic review of studies published up until 2010, found a prevalence of Alzheimer’s disease among Brazilian older adults of 11.1%. According to the authors, this figure is above the average found for other countries, but similar to rates reported in Latin America and the Caribbean (Fagundes et al., 2011).

The Specialized Component of Pharmaceutical Services is an important Brazilian strategy aimed at ensuring access to drugs provided by the Unified Health System (Sistema Único de Saúde). This strategy is characterized by providing better care to patients at ambulatory levels and its guidelines are defined by the Brazilian Ministry of Health (Brasil, 2017). It is important to mention that Brazilian guidelines are according to the international recommendation (National Institute for Health and Clinical Excellence - NICE, 2018; BMJ Best Practice, 2019).

Since 2002, drug therapies for the treatment of Alzheimer’s disease have been funded by the national public health system. These medicines include anticholinergic drug (donepezil, galantamin and rivastigmine) as first drugs of choice for treatment whereas the N-methyl-D-aspartate receptor antagonist (memantine) was included in the list of medicines provided in 2017 (Brasil, 2017). Such drugs contribute to an improvement in the clinical conditions of patients with Alzheimer’s disease with modest efficacy, but safety data that can differ. A systematic review showed an enhancement of cognitive effects for all drugs and behavioural benefits for donepezil 10 mg and galantamine 24 mg. There was a larger number of dropouts and adverse events occurring with the cholinesterase inhibitors when compared to memantine (Tan et al., 2014).

Cognitive deficit, low adherence to drug therapy and increased sensitivity to anticholinergic drugs are risk factors for adverse reactions in patients with dementia (Laroche et al., 2013). In addition, older age is frequently accompanied by polypharmacy, comorbidity and frailty (Davies and O'Mahony, 2015). Managing this scenario has proven challenging for health care systems and health policymakers (Laroche et al., 2013).

The increasing attention to drug safety and the lack of national studies to assess the risks associated with the drugs provided under the public health system for treating Alzheimer’s disease prompted this study. Patients’ reports on adverse drug reactions (ADRs) may be an important source of information for their safety and that may be unavailable from other sources (Wetzels et al., 2008; Zhu et al., 2011).

This cross-sectional study determined the prevalence and the severity of self-reported ADRs caused by the use of these medications by patients enrolled in the public health system, in a large Brazilian city.

## Methods

### Study Design

This cross-sectional study investigating ADRs to medications prescribed for the treatment of Alzheimer’s disease, self-reported by patients enrolled in the Brazilian public health system, the Unified Health System (Sistema Único de Saúde - SUS), was conducted in Sorocaba city, São Paulo state, Brazil. The Brazilian public health system comprises a number of health actions and services delivered by federal, state and municipal public organizations and institutions (Brasil, 2000).

Data were obtained from the MEDEX system and from interviews carried out with patients and/or caregivers.

### Eligibility Criteria of Study Population

Patients were considered eligible if diagnosed with International Classification of Diseases code (ICD 10): F00 (dementia in Alzheimer’s disease), F00.0 (dementia in early-onset Alzheimer’s disease), F00.1 (dementia in late-onset Alzheimer’s disease), F00.2 (dementia in Alzheimer’s disease, atypical or mixed form), F00.9 (dementia, unspecified in Alzheimer’s disease) and G30 (Alzheimer’s disease), G30.0 (early-onset Alzheimer’s disease), G30.1 (late-onset Alzheimer’s disease), G30.8 (other forms of Alzheimer’s disease), G30.9 (Alzheimer’s disease, unspecified). They were previously registered on the MEDEX system for drug dispensing and taking at least one of the following drugs: donepezil, galantamine, rivastigmine and memantine.

The interviewed subjects were the patients and/or their caregiver (aged 18 or above, considered to be involved in the daily care of the patient, regardless of being a relative or not). When the patient and caregiver were present, both were interviewed. Patients with insufficient registered data were excluded from the study.

### Study Site

Sorocaba is a major city within a metropolitan area located 92 km southeast of São Paulo city, capital of the São Paulo state, Brazil. The area of the city consists of 450,382 km^2^ and its population is approximately 671,186 (2018 estimate) (Instituto Brasileiro de Geografia e Estatística - IBGE, 2018).

The interviews were performed at the specialty drugs pharmacy. There are 40 such pharmacies in the state of São Paulo. The unit located in Sorocaba serves the patients of Sorocaba and also 48 other municipalities under the DRS–XVI (Regional Health Department – XVI). It is one of 17 departments comprising the administrative division of the Health Department for São Paulo State. It is part of Sorocaba city and it is in charge of coordinating the activities of the Department at a regional level and interfacing with municipalities and non-governmental organizations.

### Data Collection

In order to identify eligible patients, the researchers obtained data from the MEDEX system, for the period between December 2016 and April 2017. The gathered information included database registration date, health unit of origin, ICD-10 for Alzheimer’s disease and the provided drug. The enrolment status of patients on the MEDEX system is considered active while there is monthly dispensing of drugs.

The interviews with patients/caregivers were carried out at the time of patients were visiting the pharmacy to collect their medications. A pharmacy employee would notify the research team that a patient was waiting to collect their medications and the researchers would then introduce themselves. Patients that met the inclusion criteria were then invited to participate in the study. All patients/caregivers consented. All interviews took place between July and September 2017.

The questionnaire used for gathering data was developed by experts and considered the recommendation consistent across guidelines (Brasil, 2017; National Institute for Health and Clinical Excellence - NICE, 2018). There was also a pilot study testing. The researchers were trained on the proper use of scientific terminology, time spent, and the way of conducting the interviews.

The author asked patients and/or caregivers to get sociodemographic data (gender, age and assisted by caregiver or not) and the period concerning the use of the drug. The information was cross-checked with the MEDEX system. Clinical information (previous diagnosis, previous treatments, comorbidities, type of medical care, other concomitant medicines and polypharmacy) was collected too. There is no international consensus on the definition of polypharmacy. The most commonly used definition stipulates the number of concurrent drugs and the definition adopted was concerning the use of five or more medications (Laroche et al., 2013).

We also booked a second interview when the information was insufficient or when answers were not consistent. This interview was through telephone calls to the patients’ caregivers or other family members who knew about the patient’s drug treatment.

### Adverse Drug Reactions Reporting

ADR or adverse effect is any response to a medicine or medicinal product that is “noxious and unintended,” and which normally occurs in doses used in humans for the prophylaxis, diagnosis, or therapy of the disease or for the modification of physiological function (VigiAccess®, 2018).

Serious Adverse Event (SAE) is a medical occurrence that at any dose may result in death, require patient hospitalization or extension of hospitalization period. Also, it may create persistent or significant disability/incapacity, or a congenital anomaly/birth defect (VigiAccess®, 2018).

The authors collected information regarding to the self-reported of ADRs or SAEs similar to Tadesse et al. (2014). In addition, a questionnaire contained the adapted Naranjo algorithm (Pathirana et al., 2009; Lopes et al., 2014) was applied in order to establish causality for the adverse effect. The probable and definitive events were used to determine type, severity and frequency.

The characterization of ADRs was done based on the physiological system, according to the classification used by VigiBase (VigiAccess®, 2018). The treatment for Alzheimer´s disease can entail use of one medication or in association with memantine (the association commercially available in Brazil is only donepezil with memantine). Therefore, ADRs associated with drug combinations were also defined as *“ADR reported by patient and/or caregiver, but not described in medication package insert.”*

ADRs severity was categorized as: mild (not requiring specific treatment or antidotes or discontinuation of treatment); moderate (requiring change in drug therapy, and although discontinuation of treatment is unnecessary, hospitalization might be prolonged, requiring specific treatment); severe (potentially lethal, requiring suspension of the drug and specific treatment of the ADR and certainly prolonging hospitalization); and lethal (contributing directly or indirectly to patient death) (Pearson et al., 1994).

The frequency of ADRs was categorized as: very common ≥1/10 (≥10%), common ≥1/100 and <1/10 (≥1 and <10%), uncommon ≥1/1000 and <1/100 (≥0.1 and <1%), rare ≥1/10.000 and <1/1.000 (≥0.01% and <0.1%), very rare <1/10.000 (<0.01%) and unknown (described in package insert as ADR observed at post-commercialization stage but not during drug trials; these ADRs were not classified by frequency) (Meyboom and Egberts, 1999).

Reports of possible ADRs not described in the package insert were defined as *“ADR reported by patient and/or caregiver, but not described in medication package insert.”*

### Statistical Analyses

Categorical variables were expressed as absolute and relative frequencies. Proportions were compared using the Chi-square test or Fisher’s exact test. Differences were considered significant when *p* < 0.05.

To identify the characteristics of the population associated with the occurrence of ADR, we used the binary and multivariable logistic regression analysis. After the binary logistic regression analysis, the variables with *p* < 0.20 were included in the multivariable analysis.

The statistical analysis was carried out using Stata, version 12.0 and software Bioestat^®^ (version 5.3 Mamiraua Institute) software’s. Values of *p* < 0.05 were deemed statistically significant.

### Ethical Issues

This study was approved by the Research Ethics Committee of the University of Sorocaba (protocol number: 1860724). All subjects enrolled in the study were informed of the aims of the studies and signed a free and detailed consent form.

## Results

**FIGURE 1:**
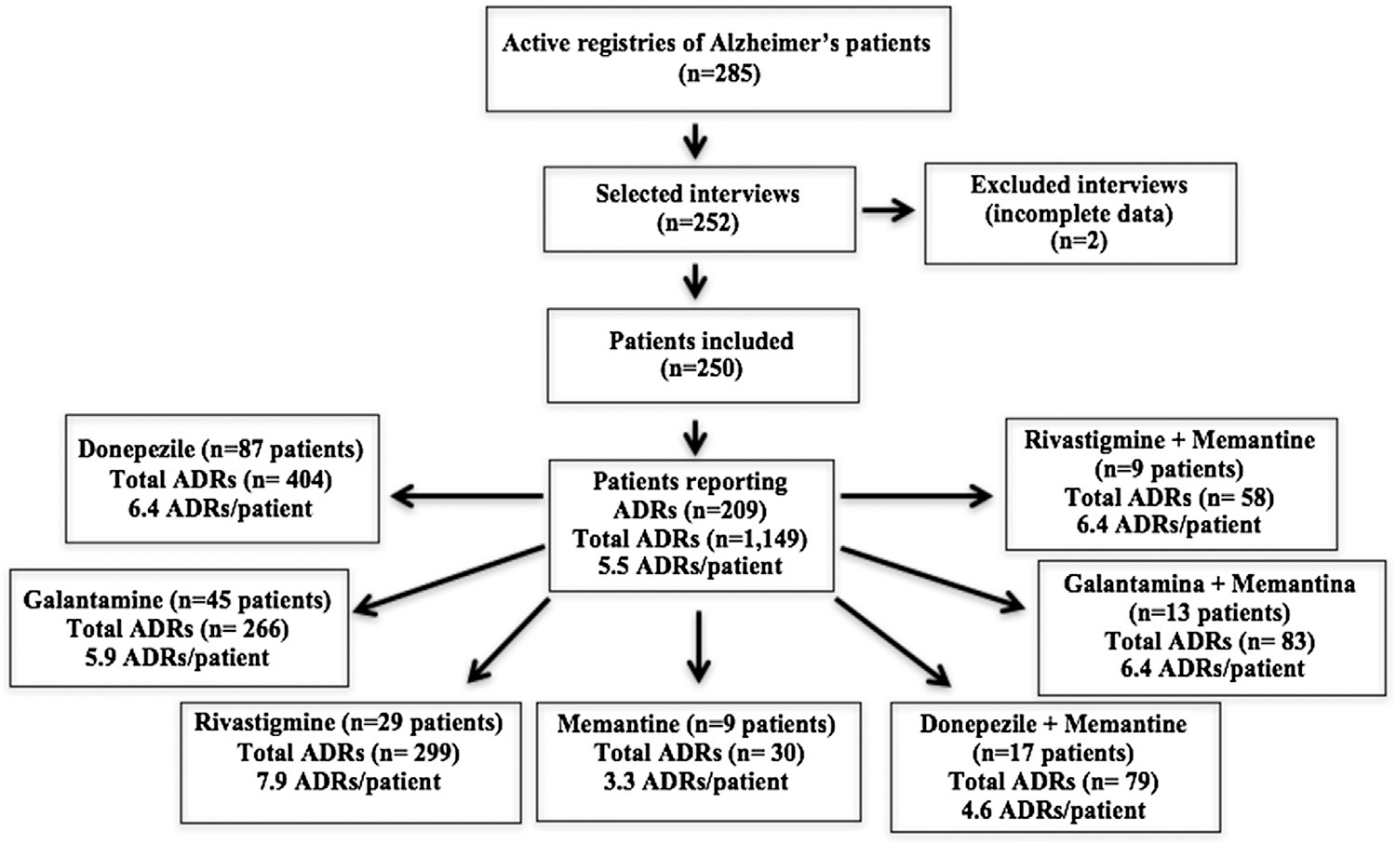
Flowchart depicting study process.

Among all 285 patients enrolled at the public pharmacy for treatment of Alzheimer’s disease, 252 (88.4%) were interviewed. Two interviews were subsequently excluded for not containing the necessary information for the study, generating a final sample of 250 patients (87.7%). Out of the total sample, 209 patients (83.6%) reported at least one possible ADR. Rivastigmine was associated with the largest number of ADRs per patient (7.9 ADRs/patient) followed by galantamine (5.9 ADRs/patient). Most patients (81.3%) used only a single medicine for treatment of Alzheimer’s disease and, on average, had more than one adverse effect related to the drugs (Figure 1).

The majority of patients were female (63.1%), aged ≥75 years (70.3%) and had been diagnosed with Alzheimer for 2–5 years (57.4%). The patients were being treated mainly with donepezil (46.1%). The drug combination was less frequent with donepezil + memantine which was used by only 8.1% of the patients. The treatment period varied between 1 and 10 years. Approximately 55.0% of the patients were assisted by a caregiver, usually a relative. A total of 70.3% of the patients suffered from comorbidities, the most cited being systemic arterial hypertension, diabetes mellitus, hypercholesterolemia and hypothyroidism (data not shown). Polypharmacy was reported in 32.5% of the assessed cases and most participants (65.5%) used the private health sector for medical visits. All 209 patients used a total of 906 drugs (average of 4.5 drug/person), with most ADRs being related to donepezil and galantamine (Table 1).

**TABLE 1:** Sociodemographic and clinical characteristics of Alzheimer’s disease patients reporting ADRs due to drug therapy (n = 209)

Variables	Donepezil 87 (41.6%) n (%)	Galantamine 45 (21.5%) n (%)	Rivastigmine 29 (13.8%) n (%)	Memantine 9 (4.3%) n (%)	Donepezil and memantine 17 (8.1%) n (%)	Galantamine and memantine 13 (6.2%) n (%)	Rivastigmine and Memantine 9 (4.3%) n (%)	Total 209 (100%) n (%)	*p*-value
Sex (n)									
Female	60 (69.0)	21 (47.0)	18 (62.0)	6 (67.0)	11 (65.0)	7 (54.0)	9 (100)	132 (63,1)	0.0002^a^
Male	27 (31.0)	24 (53.3)	11 (38.0)	3 (34.0)	6 (35.3)	6 (46.1)	0	77 (36.8)	—
Age (years)									
≤64	8 (9.2)	0	1 (3.4)	0	1 (5.9)	1 (7.7)	0	11 (5.3)	—
≥65–74	19 (22.0)	11 (24.4)	11 (38.0)	2 (22.2)	5 (29.4)	2 (15.4)	0	50 (24.0)	0.0002^a^
≥75	60 (69.0)	34 (75.5)	17 (58.6)	7 (77.7)	11 (5.9)	10 (77.0)	9 (100.0)	148 (71.0)	—
Not stated	0	0	0	0	0	0	0	0	—
Time of diagnosis of disease (years)									
0‒2	8 (9.2)	2 (4.4)	4 (14.0)	1 (11.1)	1 (5.9)	0	0	16 (7.6)	<0.0001^a^
>2‒5	55 (63.2)	27 (60.0)	10 (34.6)	3 (33.3)	9 (52.9)	10 (77.0)	4 (44.4)	118 (56.4)	
>5‒10	20 (23.0)	13 (29.0)	10 (34.4)	3 (34.0)	6 (35.3)	3 (23.0)	5 (55.5)	60 (28.7)	
>10	3 (3.4)	1 (2.2)	3 (10.3)	0	1 (5.9)	0	0	8 (3.8)	
Not stated	1 (1.1)	2 (4.4)	2 (6.9)	2 (22.2)	0	0	0	7 (3.3)	
Time of use of drug (years)									
0‒1	12 (13.8)	5 (11.1)	4 (13.8)	2 (22.2)	1 (5.9)	0	1 (11.1)	25 (12.0)	<0.0001^a^
>1‒2	21 (24.1)	11 (24.4)	4 (13.8)	2 (22.2)	5 (29.4)	4 (30.7)	0	47 (22.5)	
>2‒3	20 (23.0)	2 (4.4)	2 (6.9)	1 (11.1)	2 (11.7)	2 (15.4)	2 (22.2)	31 (15.0)	
>3‒5	13 (15.0)	13 (29.0)	4 (13.8)	2 (22.2)	4 (23.5)	3 (23.0)	1 (11.1)	40 (19.1)	
>5‒10	14 (16.0)	10 (22.2)	9 (31.0)	2 (22.2)	3 (17.6)	2 (15.4)	4 (44.4)	44 (21.0)	
>10 or more	3 (3.4)	3 (6.6)	4 (13.8)	0	1 (5.9)	0	0	11 (5.3)	
Not stated	4 (4.1)	1 (2.2)	2 (6.9)	0	1 (5.9)	2 (15.4)	1 (11.1)	11 (5.3)	
Caregiver (n)									
None	14 (16.1)	3 (6.6)	5 (17.2)	0	0	0	0	22 (10.5)	<0.0001^a^
1	55 (63.2)	22 (49.0)	15 (52.0)	1 (11.1)	11 (65.0)	9 (69.2)	3 (33.3)	116 (55.5)	
2–4	13 (16.0)	16 (35.6)	6 (20.8)	2 (22.2)	4 (23.5)	2 (15.4)	5 (55.5)	49 (23.4)	
>4	3 (3.4)	1 (2.2)	0	4 (44.4)	1 (5.9)	2 (15.4)	0	11 (5.3)	
Not stated	2 (2.2)	3 (6.6)	3 (10.3)	2 (22.2)	1 (5.9)	0	1 (11.1)	11 (5.3)	
Medical care									
Public	55 (63.2)	14 (31.1)	8 (27.6)	1 (11.1)	3 (17.6)	2 (15.4)	1 (11.1)	57 (27.3)	<0.0001^a^
Private	4 (4.6)	28 (62.2)	18 (62.0)	6 (66.7)	13 (76.5)	11 (84.6)	6 (66.7)	137 (65.5)	
Not stated	—	3 (6.7)	3 (10.3)	2 (22.2)	1 (5.9)	0	2 (22.2)	15 (7.2)	
Comorbidities									
Yes	68 (78.2)	31 (69.0)	21 (72.4)	5 (55.5)	9 (53.0)	8 (61.5)	5 (55.5)	147 (70.3)	<0.0001^a^
No	11 (12.6)	11 (24.4)	2 (6.9)	0	3 (17.6)	1 (7.7)	0	17 (8.1)	
Not stated	—	—	6 (20.6)	4 (44.4)	5 (29.4)	4 (30.7)	4 (44.4)	45 (2.5)	
Comorbidities (n)	138	66	52	18	21	14	12	321	
Polypharmacy#									
Yes	15 (17.2)	13 (29.0)	11 (38.0)	8 (89.0)	9 (53.0)	6 (46.1)	6 (67.0)	68 (32.5)	<0.0001^a^
No	54 (62.0)	27 (60.0)	17 (58.6)	1 (11.1)	8 (47.0)	7 (54.0)	3 (333)	117 (56.0)	
Not stated	18 (20.7)	5 (11.1)	1 (3.4)	0	0	0	0	24 (11.5)	
Medications (n)	224	194	142	70	101	65	68	874	

n, number. # Polypharmacy, use of five or more medications.

a Statistically significant difference on Chi-square test, *p* < 0.05.

The most widely used drugs were to treat diseases of the cardiovascular system (33.0%) and central nervous system (25.0%) being mainly: selective serotonin reuptake inhibitors (ATC: N06AB); Beta blocking agents, non-selective (ATC: C07AA); angiotensin II receptor blockers (ATC: C09CA) and diazepines, oxazepines, thiazepines and oxepines (ATC: N05AH) (data not show).

According to causality score, the events related to the Alzheimer drugs classified as probable or definitive were shown in Tables 2–4.

**TABLE 2:** Prevalence of self-reported adverse effects to medications used for treatment of Alzheimer’s disease according to physiological system and frequency (n = 1,149)

Physiological systems	Donepezil	Galantamine	Rivastigmine	Memantine	Donepezil + memantine	Galantamine + memantine	Rivastigmine + memantine	Total
Central nervous system disorders	103 (25.5)	68 (25.5)	58 (25.3)	6 (20.7)	19 (24.0)	27 (32.5)	13 (22.4)	294
Dizziness	40 (10.0)^b^	0	14 (6.1)^a^	0	4 (5.0)^a^	2 (2.4)^g^	2 (3.4)^g^	62
Syncope	10 (2.5)^b^	3 (1.1)^b^	2 (0.9)^c^	0	1 (1.3)^g^	0	0	16
Convulsion	3 (0.7)^f^	2 (0.7)^e^	2 (0.9)^d^	1 (3.4)^e^	3 (3.8)^g^	0	0	11
Headache	31 (7.7)^a^	10 (3.7)^b^	16 (7.0)^b^	1 (3.4)^b^	4 (5.0)^g^	1 (1.2)^g^	4 (6.9)^g^	67
Tremor	0	9 (3.4)^b^	12 (5.2)^b^	0	0	2 (2.4)^g^	3 (5.2)^g^	26
Somnolence	2 (0.5)^g^	36 (13.5)^b^	9 (0.9)^b^	0	2 (2.5)^g^	13 (15.7)^g^	3 (5.2)^g^	65
Lethargy	0	4 (1.5)^b^	0	0	0	3 (3.6)^g^	0	7
Balance disorders	17 (4.2)^b^	4 (1.5)^b^	3 (1.3)^c^	4 (13.8)^g^	5 (6.3)^g^	6 (7.2)^g^	1 (1.7)^g^	40
General disorders	19 (4.7)	27 (10.1)	24 (10.5)	6 (20.7)	4 (5.0)	11 (13.2)	3 (5.2)	94
Asthenia	0	1 (0.4)^b^	0 (0)	0	0	1 (1.2)^g^	0	2
Fatigue (tiredness)	19 (4.7)^b^	9 (3.4)^b^	20 (8.7)^b^	2 (6.9)^c^	3 (3.8)^g^	5 (6.0)^g^	2 (3.4)^g^	60
Malaise	0	16 (6.0)^b^	2 (0.9)^b^	0	0	4 (4.8)^g^	0	22
Ataxia	0	1 (0.4)^g^	2 (0.9)^b^	4 (13.8)^c^	1 (1.3)^g^	1 (1.2)^g^	1 (1.7)^g^	10
Gastrointestinal disorders	52 (12.9)	49 (18.4)	24 (10.5)	2 (6.9)	10 (12.6)	8 (9.6)	6 (10.3)	151
Nausea	12 (2.9)^a^	13 (4.9)^a^	9 (0.9)^a^	0	3 (3.8)^g^	2 (2.4)^g^	3 (5.2)^g^	42
Vomiting	8 (2.0)^b^	12 (4.5)^a^	4 (1.7)^a^	0	1 (1.3)^g^	0	0	25
Abdominal discomfort	3 (0.7)^b^	11 (4.1)^b^	5 (2.2)^b^	2 (6.9)^g^	3 (3.8)^g^	3 (3.6)^g^	1 (1.7)^g^	28
Diarrhea	28 (7.0)^a^	8 (3.0)^b^	6 (2.6)^a^	0	3 (3.8)^a^	2 (2.4)^g^	2 (3.4)^g^	49
Dyspepsia	1 (0.2)^g^	5 (1.9)^c^	0	0	0	1 (1.2)^g^	0	7
Psychiatric disorders	152 (37.6)	37 (14.0)	93 (40.6)	14 (48.3)	34 (43.0)	19 (22.9)	30 (51.7)	379
Hallucinations	22 (5.4)^f^	19 (7.1)^e^	6 (2.6)^f^	6 (20.7)^c^	5 (6.3)^g^	11 (13.2)^g^	4 (6.9)^g^	73
Agitation	34 (8.4)^f^	1 (0.4)^g^	18 (7.8)^b^	0	10 (12.6)^a^	0	6 (10.3)^g^	69
Aggression	20 (5.0)^g^	0	1 (0.4)^f^	0	8 (10.1)^g^	0	0	29
Anxiety	0	0	20 (8.7)^b^	0	0	0	5 (8.6)^g^	25
Depression	1 (0.2)^g^	17 (6.4)^b^	8 (3.5)^c^	0	0	6 (7.2)^G^	3 (5.2)^g^	35
Insomnia	39 (9.6)^b^	0	10 (4.4)^c^	0	4 (5.0)^g^	1 (1.2)^g^	2 (3.4)^g^	56
Mental confusion	10 (2.5)^g^	0	21 (9.2)^b^	8 (27.6)^c^	2 (2.5)^a^	1 (1.2)^g^	8 (13.8)^g^	50
Sleep disorders	26 (6.4)^b^	0	9 (4.0)^g^	0	5 (6.3)^g^	0	2 (3.4)^g^	42
Heart diseases	2 (0.5)	4 (1.5)	0	0	1 (1.3)	1 (1.2)	0	6
Bradycardia	2 (0.5)^g^	1 (0.4)^b^	0	0	1 (1.3)^g^	1 (1.2)^g^	0	5
Palpitation	0	3 (1.1)^c^	0	0	0	0	0	3
Metabolism and nutrition disorders	4 (1.0)	37 (14.0)	10 (4.3)	0	1 (1.3)	11 (13.2)	1 (1.7)	64
Decreased appetite	0	16 (6.0)^b^	6 (2.6)^a^	0	0	2 (2.4)^g^	0	24
Dehydration	0	7 (2.6)^c^	1 (0.4)^f^	0	0	3 (3.6)^g^	1 (1.7)^g^	12
Weight loss	1 (0.2)^g^	14 (5.2)^b^	3 (1.3)^b^	0	0	4 (4.8)^g^	0	22
Anorexia	3 (0.7)^b^	0	0	0	1 (1.3)^g^	2 (2.4)^g^	0	6
Skin and subcutaneous tissue disorders	1 (0.2)	1	3 (1.3)	0	0	0	2 (3.4)	7
Hyperhidrosis	1 (0.2)^g^	1 (0.4)^c^	0	0	0	0	1 (1.7)^g^	3
Pruritus (itching)	0	0	3 (1.3)^d^	0	0	0	1 (1.7)^g^	4
Vascular disorders	0	1 (0.4)	0	1 (3.4)	1 (1.3)	0	1 (1.7)	4
Hypertension	0	0	0	1 (3.4)^b^	1 (1.3)^g^	0	1 (1.7)^g^	3
Hypotension	0	1 (0.4)^c^	0	0	0	0	0	1
Musculoskeletal disorders	44 (10.9)	22 (8.3)	16 (7.0)	0	4 (5.0)	4 (4.8)	0	90
Muscle spasms	24 (6.0)^b^	5 (1.9)^b^	0	0	3 (3.8)^g^	0	0	32
Myalgia	18 (4.5)^b^	4 (1.5)^g^	7 (3.0)^b^	0	0	0	0	29
Muscle weakness	1 (0.2)^g^	13 (4.9)^c^	9 (4.0)^b^	0	0	4 (4.8)^g^	0	27
Rhabdomyolysis	1 (0.2)^g^	0	0	0	1 (1.3)^g^	0	0	2
Respiratory diseases	10 (2.5%)	2 (0.7)	0	0	1 (1.3)	0	1 (1.7)	14
Dyspnea	0	1 (0.4)^g^	0	0	0	0	1 (1.7)^g^	2
Hiccups	0	1 (0.4)^g^	0	0	0	0	0	1
Common cold	10 (2.5)^b^	0	0	0	1 (1.3)^a^	0	0	11
Infections and infestations	15 (3.7)	0	0	1 (3.4)	4 (5.0)	0	0	20
Urinary tract infection	14 (3.5)^g^	0	0	1 (3.4)^g^	4 (5.0)^a^	0	0	19
Nasopharyngitis	1 (0.2)^g^	0	0	0	0	0	0	1
Hepatobiliary disorders	0	11 (4.1)	1 (0.4)	0	0	2 (2.4)	1 (1.7)	15
Hepatitis	0	2 (0.7)^e^	1 (0.4)^g^	0	0	0	1 (1.7)^g^	4
Blurred vision	0	9 (3.4)^c^	0	0	0	2 (2.4)^g^	0	11
Ear and inner ear disorders	2 (0.5)	7 (2.6)	0	0	0	0	0	9
Vertigo	2 (0.5)^g^	6 (2.2)^b^	0	0	0	0	0	8
Dizziness	0	1 (0.4)^e^	0	0	0	0	0	1
Total ADRs	404 (100.0)	266 (100.0)	229 (100.0)	30 (100.0)	79 (100.0)	83 (100.0)	58 (100.0)	1,149

ADR, adverse drug reaction.

a Very common (10% frequency)^.^

b Common (1–10% frequency)

c Uncommon (0.1–1% frequency).

d Rare (0.01–0.1% frequency)^.^

e Very rare (frequency <0.01%)^.^

f Unknown (described on medication package insert as adverse effect seen only at post-commercialization stages, not classified for frequency).

g ADR reported by patient and/or caregiver, but not described in medication package insert.

Physiological system has been classified according to VigiBase.

**TABLE 3:** Prevalence of self-reported adverse effects to medications commonly used for treating Alzheimer disease according to frequency (n = 1,149).

ADR frequency	Donepezil n (%)	Galantamine n (%)	Rivastigmine n (%)	Memantine n (%)	Donepezil and memantine n (%)	Galantamine and memantine n (%)	Rivastigmine and memantine n (%)	Total n (%)
Very common^a^	71 (17.6)	25 (9.4)	39 (17.0)	0	24 (30.4)	0	0	159 (13.8)
Common^b^	217 (54.0)	170 (64.0)	144 (62.9)	2 (6.7)	0	0	0	533 (46.3)
Uncommon^c^	0	39 (14.7)	23 (10.0)	20 (66.7)	0	0	0	82 (7.1)
Rare^d^	0	0	5 (2.2)	0	0	0	0	5 (0.4)
Very rare^e^	0	24 (9.0)	6 (2.6)	1 (3.3)	0	0	0	31 (2.7)
Unknown^f^	59 (14.6)	0	2 (0.8)	0	0	0	0	61 (5.3)
Not described in medication package insert^g^	57 (14.1)	8 (3.0)	10 (4.4)	7 (23.3)	55 (69.6)	83 (100.0)	58 (100.0)	278 (24.2)
Total	404 (100.0)	266 (100.0)	229 (100.0)	30 (100.0)	79 (100.0)	83 (100.0)	58 (100.0)	1149 (100.0)

ADR, adverse drug reaction.

a Very common (10% frequency).

b Common (1–10% frequency).

c Uncommon (0.1–1% frequency).

d Rare (0.01–0.1% frequency).

e Very rare (<0.01%).

f Unknown (described in medication package insert as adverse effect seen only at post-commercialization stages, not classified for frequency).

g ADR reported by patient or caregiver, but not described in medication package insert.

**TABLE 4:** Clinical aspects of Alzheimer’s disease patients and self-reported ADRs (n = 209).

Variables	Donepezil (n = 87) n (%)	Galantamine (n = 45) n (%)	Rivastigmine (n = 29) n (%)	Memantine (n = 9) n (%)	Donepezil and memantine (n = 17) n (%)	Galantamine and memantine (n = 13) n (%)	Rivastigmine and memantine (n = 9) n (%)	Total n (%)	*p*-value
Individual identifying adverse effect									
Patient	7 (8.0)	2 (4.4)	4 (13.7)	0	0	0	1 (11.1)	14 (6.7)	tbl4fnlowast < 0.0001^a^
Caregiver	74 (85.0)	40 (88.9)	24 (82.7)	4 (44.4)	16 (94.1)	12 (92.3)	7 (77.7)	177 (56.0)	
Health professional	6 (6.8)	3 (6.6)	1 (3.4)	5 (55.5)	1 (5.9)	1 (7.7)	1 (11.1)	18 (8.8)	
Adverse effect severity									
Mild	79 (91.0)	36 (80.0)	25 (86.2)	9 (100.0)	16 (94.1)	12 (92.3)	9 (100.0)	186 (89.0)	tbl4fnlowast < 0.0001^a^
Moderate	7 (8.0)	4 (8.9)	4 (13.7)	0	1 (5.9)	1 (7.7)	0	17 (8.1)	
Severe	1 (1.1)	5 (11.1)	0	0	0	0	0	6 (2.9)	
Adverse effect									
Discontinuation or switch of medication	9 (10.3)	4 (8.9)	4 (13.7)	0	1 (5.9)	1 (7.7)	0	19 (9.1)	tbl4fnlowast < 0.0001^a^
Hospitalization	1 (1.1)	5 (11.1)	0	0	0	0	0	6 (2.9)	
No change	77 (88.5)	36 (80.0)	25 (86.2)	9 (100.0)	16 (94.1)	12 (92.3)	9 (100.0)	184 (88.0)	

a Statistically significant difference on Chi-square test, *p* < 0.05.

Table 2 describes possible ADRs according to physiological systems and frequency. Effects were reported predominantly for donepezil and galantamine and mainly related to psychiatric disorders, being 25.5% for donepezil and 25.5% for galantamine. The most commonly reported effects for donepezil were agitation, vertigo, insomnia and headaches (considered common or very common). The most commonly reported effects for galantamine were somnolence, depression, malaise (considered common) and hallucinations (rare). Confusion, anxiety, agitation and headaches were the effects most commonly reported for rivastigmine and also considered common.

Among all 1,149 ADRs reported, most were classified as “common” (46.3%), 5.3% were of unknown frequency and approximately 24% of ADRs were not described in the medication package insert (there were patient/caregiver reports of the effect, but this was not described in the medication package insert). No uncommon ADRs were reported for donepezil, but for galantamine, rivastigmine and memantine, 7.1% of ADRs were classified as “uncommon.” Galantamine presented the most “very rare” ADRs, although the total number of these reactions was very small (Table 3).

Table 4 describes the reported ADRs, adequate measures to handle them and their classification according to severity. The caregiver is the individual that most frequently recognized certain symptoms as ADRs (56.0%). Most ADRs were considered of mild severity (89.0%). Six patients (2.4%) reported SAEs leading to hospitalization (galantamine n = 5 and donepezil n = 1), which involved nausea, vomiting, abdominal discomfort, diarrhea, dehydration and syncope.

In Table 5, we showed the crude and adjusted analyses, which aimed at identifying patients’ characteristics regardless of the association with ADR. After the adjusted analyses, the use of antipsychotics was the variable associated with ADR (Odds Ratio = 4.95; 95% CI: 1.45–16.93; *p* = 0.011).

**TABLE 5:** Crude and adjusted analyses of adverse drug reactions (ADR) according to demographic and health variables and polypharmacy in patients with Alzheimer´s disease (n = 244)

Variables	ADR (%)	Crude analyses OR (95%CI)	*p* value^a^	Adjusted analyses OR (95%CI)	*p* value^b^
Sex	—	—	0.522	—	—
Male	87.1	1.00	—	—	—
Female	84.0	0.78 (0.36; 1.67)	—	—	—
Age (years)	—	—	0.124	—	0.127
≤59	100.0	1.00	—	1.00	—
60–74	90.2	1.80 (0.71; 4.59)	—	1.80 (0.71; 4.59)	—
≥75	83.5	0.83 (0.34; 2.04)	—	0.83 (0.34; 2.04)	—
Diabetes mellitus	—	—	0.789	—	—
No	82.9	1.00	—	—	—
Yes	84.4	1.12 (0.50; 2.48)	—	—	—
Hypertension	—	—	0.458	—	—
No	85.4	1.00	—	—	—
Yes	81.6	076 (0.36; 1.58)	—	—	—
Dyslipidemia	—	—	0.110	—	0.107
No	85.8	1.00	—	1.00	—
Yes	76.4	0.53 (0.25; 1.15)	—	0.52 (0.24; 1.15)	—
Depression	—	—	0.852	—	—
No	82.9	1.00	—	—	—
Yes	83.9	1.07 (0.52; 2.23)	—	—	—
Hypothyroidism	—	—	0.197	—	0.452
No	85.0	1.00	—	1.00	—
Yes	76.7	0.58 (0.25; 1.33)	—	0.71 (0.29; 1.73)	—
Osteoporosis	—	—	0.304	—	—
No	84.1	1.00	—	—	—
Yes	75.0	0.56 (0.19; 1.67)	—	—	—
Time of treatment (years)	—	—	0.848	—	—
0 to 1	79.3	1.00	—	—	—
> 1 to 3	89.7	2.26 (0.73; 7.02)	—	—	—
> 3 or more	82.3	1.26 (0.45; 3.52)	—	—	—
Drugs for AD	—	—	0.623	—	—
Memantine	75.0	1,00	—	—	—
Donepezil	85.4	1.96 (0.47; 8.06)	—	—	—
Galantamine	84.9	1.88 (0.41; 8.47)	—	—	—
Rivastigmine	87.9	2.42 (0.45; 12.88)	—	—	—
Donepezil + memantine	84.2	1.78 (0.29; 10.72)	—	—	—
Galantamine + memantine	85.7	2.00 (0.27; 14.59)	—	—	—
Rivastigmine + memantine	100.0	1.00 (0.51; 3.46)	—	—	—
Donepezil + rivastigmine	50.0	0.33 (0.02; 7.14)	—	—	—
Use of antipsychotics	—	—	0.011	—	0.011
No	79.0	1.00	—	1.00	—
Yes	94.8	4.88 (1.43; 16.65)	—	4.95 (1.45; 16.93)	—
Polypharmacy	—	—	0.327^a^	—	—
No	81.6	1.00	—	—	—
Yes	87.0	1.50 (0.66; 3.42)	—	—	—

a Logistic binary regression.

b Multivariate logistic regression

AD, Alzheimer´s disease; OR, adds ratio.

## Discussion

### Summary of Evidence and Comparison to the Findings of Previous Studies

The study comprises a sample of patients with Alzheimer’s disease who were users of the Brazilian public health system in the city of Sorocaba, State of São Paulo. The assessed population was predominantly female, aged 75 or older, suffered from other comorbidities and required at least one caregiver (usually a family member). Also, the patients were unable to take medications without assistance and used the private health sector for most visits.

National scientific literature has also shown that females are more likely to develop Alzheimer’s disease than males (Fagundes et al., 2011; Barros De Matos and Decesaro, 2012). A study showed that postmenopausal females, between 40 and 60 years old, when compared to premenopausal females, had lower levels of glucose in the brain and also exhibited higher levels of mitochondrial dysfunction. This phenomenon might cause diminished energy processing and reduced memory, which can be linked to dementia (Fagundes et al., 2011).

Almost 70% of patients suffered from at least one other disease; this might be due to the advanced age of the assessed subjects. Among the reported comorbidities, systemic arterial hypertension, diabetes, hypothyroidism and hypercholesterolemia were the most frequent ones. According to the scientific literature, hypertension, dyslipidemias, hyperinsulinemias, type 2 diabetes, obesity, atherosclerosis and arrhythmias are associated with increased risk for cognitive deficit, dementia and Alzheimer’s disease (Kalaria et al., 2008). A study involving 130 older Brazilian adults with this disease discovered that 75% of these individuals also suffered from another disease, with the main prevalence of hypertension (53.3%) and diabetes mellitus (21.7%) (Barros De Matos and Decesaro, 2012).

Although patients collected their medications from the public health system, most of them used the private sector for medical appointments and exams. This may be due to the high cost of these medications (Brasil, 2013) and to the fact that the private health sector is a faster option for medical treatment, more readily providing the exams required to meet the criteria determined by the national health services.

The most used medications were donepezil and galantamine. Most of the patients (83.5%) reported at least one ADR which involved mainly donepezil and galantamine although the largest number of ADRs per patient referred to rivastigmine (7.9 ADRs per patient) and galantamine (5.9 ADRs per patient). There was a small number of prescriptions for memantine due to the fact that this drug was only available through the public health system in the year of 2017, which coincided with the year of the data collection.

In general, mild severity and common frequency were the adverse effects. The reported effects were mostly associated with psychiatric disorders, insomnia, agitation, and sleep disorders being the most commonly reported effect. Although hallucination was considered a rare effect, it was reported by patients that used galantamine. Among the treatments for the most common diseases in the patients who were studied, adjusted analyses showed that the use of antipsychotics was the variable associated with ADR.

The use of galantamine, rivastigmine and memantine was associated with rare or very rare ADRs, accounting for 3.8% of all samples and mainly due to galantamine. Approximately 2.5% of SAEs resulted in hospitalization due to the use of galantamine (n = 5 patients) and donepezil (n = 1 patient), leading to gastrointestinal complications which involved nausea, vomiting, abdominal discomfort, diarrhoea, dehydration and syncope.

The findings of this study corroborate data from the VigiBase database, an international drug monitoring program created by the World Health Organization, showing a larger number of suspected ADRs caused by donepezil and rivastigmine (VigiAccess®, 2018). The main ADR reports described for donepezil (agitation, vertigo, insomnia and headache), galantamine (somnolence, depression and malaise), and rivastigmine (mental confusion, hallucination, ataxia and impaired balance) are also among the most commonly reported reactions in the VigiBase database (VigiAccess®, 2018).

Effects were predominantly reported for donepezil and galantamine and mainly related to psychiatric disorders, being 25.5% for donepezil and 25.5% for galantamine. The most commonly reported effects for donepezil were agitation, vertigo, insomnia and headaches (with common or very common frequency). The most commonly reported effects for galantamine were somnolence, depression, malaise (considered common) and hallucinations (rare). Confusion, anxiety, agitation and headaches were the effects most commonly reported for rivastigmine and also considered of common frequency. The main ADR reports described for donepezil, galantamine and rivastigmine were also the most commonly reported reactions in the VigiBase database (VigiAccess®, 2018).

An observational study collected information from a French database of spontaneous notification of adverse events related to the use of anticholinergic drug. Although the population in this study does not represent the population with Alzheimer’s disease, it is noteworthy that around 31% (n = 118) of the observed drug interactions were responsible for ADRs that involved mainly the cardiovascular system (bradycardia, atrioventricular block and hypotension) and central nervous system (mainly causing mental confusion) (Tavassoli et al., 2007). Future studies may consider collecting information regarding the role of drug interactions in causing ADRs in the population with Alzheimer’s disease or other dementias.

Another study analyzed, for a period of 16 years (1998–2013), the ADRs registered in VigiBase (referring to 58 countries and five continents) related to the use of anticholinesterase drugs for the treatment of Alzheimer’s disease. Rivastigmine and donepezil were involved in most reports and occurred mainly due to neuropsychiatric disorders, which is similar to the findings of our study. However, they noticed that serious ADRs were more often reported than nonserious, which differs from our findings (Kröger et al., 2015).

A survey analyzed two national pharmacovigilance databases (Food and Drug Administration Adverse Event Reporting System - FAERS and the Canada Vigilance Adverse Reaction Database - CVARD) concerning the adverse effects of cholinesterase inhibitors in dementia, between 2004 and 2012. There is higher frequency of reports of death and serious adverse events for rivastigmine when compared to other drugs (FAERS, odds ratio = 3.42 and CVARD, odds ratio = 3.67 databases) (Ali et al., 2015).

Our results differed from the findings of Kröger et al. (2015) and Ali et al. (2015) since the ADRs in our study were mainly of mild severity. It is important to mention that there is a limited number of studies related to the topic in the literature, and the studies that were found refer to spontaneous notification of ADRs, which differ from the present study that performed the active search for them.

### Strengths and Limitations of This Study

In the present study, patients could have difficulties remembering the name of diseases, medications currently being used, and other information. Thus, some relevant data may have been missed, as they had not been registered in the MEDEX system. In order to minimize the effects of this limitation, missing information was substituted with data found in medical prescriptions and gathered through new interviews. Other limitation of the MEDEX system is lack of classification concerning the stages or types of dementia (early or late stages and atypical or mixed form, among others).

It is important to emphasize that the findings of this study are limited to individuals with Alzheimer´s disease whose medications were provided by the public health system, and did not include patients who paid for their treatment. Although the data cannot be generalized due to the fact that they are restricted to the population of a city and of a health public sector, lack of information regarding this population reinforces the need of this study.

The fact that the subjects self-reported the ADRs might represent a limitation, as other diseases that affect the older adults can often cause similar symptoms to those self-reported, which may have led to overestimation of ADR prevalence. On the other hand, when the patient and caregiver report the adverse effect and/or when the report is done during the use of the drug, there is an increase in the confidence of these findings and a decrease in the recall bias. In addition, the use of modified Naranjo algorithm, to standardize the causality estimate, conferred greater confidence in the report.

There is insufficient available data in the scientific literature regarding the prevalence of ADRs in patients undergoing treatment for Alzheimer’s disease, which makes the preliminary data, described in this study, useful in helping the public health service to better manage this problem.

In the assessed period, the sample comprised 88% of the whole population suffering from Alzheimer’s disease with an active status, allowing the patients to obtain medicines from the Specialty Drug Pharmacy of the city of Sorocaba. Also, the instrument used for data gathering was carefully developed, and a pilot study test was also carried out. The researchers involved in the study had been previously trained to better use terminology and conduct interviews.

### Implications for Clinical Practice and for Research

Although the cholinergic effects of these drugs may contribute to the incidence of neuropsychiatric events, it is important to note that patients suffering from Alzheimer’s disease are likely to experience neuropsychiatric symptoms as part of the disease process and psychotropic use may further increase this likelihood (Gustafsson et al., 2013). In fact, in the present study, the use of antipsychotics increased the risk of ADR in these patients.

It is noteworthy that results from clinical exams for identifying ADRs are usually unspecific, and more than one organ is often involved, which makes it difficult to precisely identify ADRs. There is also a chance of a possible iatrogenic cascade, which can increase treatment costs, when signs and symptoms caused by ADRs are treated as a new disease (Fonteles et al., 2009). In older adult patients, this situation is even more critical due to other comorbidities associated with aging (Varallo et al., 2010).

Despite the large number of ADRs reported, the majority of them were classified as mild severity and resolved without the need for intervention of health professionals or hospitalization, or constituting an emergency. There was a total of six reported ADRs that were considered severe requiring hospitalization, largely due to the use of galantamine.

Given that projections indicate an increase in Alzheimer’s disease cases, the demand for these medications is set to rise in the upcoming years. Clinical protocols could better address the follow-up of patients undergoing drug therapy for Alzheimer’s disease. Such procedures could allow early identification of therapeutic failure and facilitate handling of ADRs. The findings of this study can help guide public health administrators, drug prescribers, caregivers and patients through safety aspects and improve care regarding the drug treatment of patients with Alzheimer’s disease.

In light of insufficient data on this issue in the scientific literature, more primary studies on drug treatment of Alzheimer’s disease in the Brazilian population should be encouraged.

## Conclusion

The results revealed a large number of ADRs used in the treatment of Alzheimer’s disease, which were generally classified as being of common frequency and mild severity. Donepezil had the largest number of reported ADRs. The most prevalent adverse effects were psychiatric disorders.

In this way, before starting a treatment for neuropsychiatric symptom, it is essential to verify if it is not a result of the use of these drugs. Improving safety-related care in the use of drugs for treating Alzheimer’s disease is extremely important when taking into account fragility, the use of a larger number of drugs, and comorbidities in patients.

## Data Availability Statement

The datasets generated for this study are available on request to the corresponding author.

## Ethics Statement

The studies involving human participants were reviewed and approved by Research Ethics Committee of the University of Sorocaba (protocol number: 1860724). The patients/participants provided their written informed consent to participate in this study.

## Author Contributions

TF is the principal investigator, participated in all stages of the study and writhed the manuscript. CB is the project manager and co-investigator, was involved in study selection and data analysis, and contributed to the writing and revision of the manuscript. LL is co-investigator, contributed to the writing and revision of the manuscript. All authors read and approved the final manuscript.

## Conflict of Interest

The authors declare that the research was conducted in the absence of any commercial or financial relationships that could be construed as a potential conflict of interest.
